# The Benefit of Intraoperative Magnetic Resonance Imaging in Endoscopic and Microscopic Transsphenoidal Resection of Recurrent Pituitary Adenomas

**DOI:** 10.3390/curroncol29010035

**Published:** 2022-01-17

**Authors:** Andrej Pala, Andreas Knoll, Max Schneider, Gwendolin Etzrodt-Walter, Georg Karpel-Massler, Christian Rainer Wirtz, Michal Hlavac

**Affiliations:** 1 Department of Neurosurgery, University of Ulm, Lindenallee 2, 89312 Günzburg, Germany; max.schneider@uni-ulm.de (M.S.); rainer.wirtz@bkh-guenzburg.de (C.R.W.); michal.hlavac@uni-ulm.de (M.H.); 2 Department of Neurosurgery, University of Ulm, Alber-Einsteint-Allee 23, 89081 Ulm, Germany; andreas.knoll@uni-ulm.de (A.K.); georg.karpel-massler@uniklinikum-ulm.de (G.K.-M.); 3 Department of Endokrinology, Endokrinologiezentrum Ulm, Bahnhofplatz 7, 89073 Ulm, Germany; etzrodt-walter@endokrinologie-ulm.de

**Keywords:** transsphenoidal surgery, intraoperative MRI, pituitary adenoma, intraoperative tumor remnant, complications

## Abstract

The surgical treatment of recurrent adenomas can be challenging. Intraoperative magnetic resonance imaging (iMRI) can improve the orientation and increase the safe extent of resection. We conducted a quantitative and qualitative retrospective analysis of recurrent adenomas treated by endoscopic or microscopic iMRI-assisted transsphenoidal surgery. A total number of 59 resections were selected. Detailed volumetric measurements, tumor characteristics, and MRI features of intraoperative remnants were evaluated. Intraoperative MRI increased the gross total resection (GTR) rate from 33.9% to 49.2%. Common locations of tumor remnants after iMRI were the clivus, the wall of the cavernous sinus or the perforation of the diaphragm. Increasing tumor volume and the microscopic technique were significantly associated with further resection after iMRI in the univariate analysis (*p* = 0.004, OR 1.6; *p* = 0.009, OR 4.4). Only the increasing tumor volume was an independent predictor for further resection (*p* = 0.007, OR 1.5). A significantly higher proportion of GTRs was achieved with the endoscopic technique (*p* = 0.001). Patients with a large recurrent pituitary adenoma who underwent microscopic transsphenoidal resection were the most likely to benefit from iMRI regarding the extent of resection. Occult invasions of the cavernous sinus and/or the clivus were the most common findings leading to further resection of tumor remnants after iMRI.

## 1. Introduction

Recurrent pituitary adenomas are benign tumors, but based on their potentially aggressive growth and infiltration of adjacent structures, treatment may be challenging. Moreover, pituitary adenomas mostly occur in middle-aged patients with a long life expectancy. The extent of resection (EOR) is an important prognostic marker for progression-free survival (PFS) in various intracranial pathologies [1,2]. Similarly, gross total resection (GTR) has been shown to have a positive impact on PFS [1,2]. In the case of adenoma recurrence, surgical resection is the most common treatment step. However, due to fibrosis and different attributes of the tissue after primary resection, the anatomical borders and normal planes may be difficult to dissect [3]. Intraoperative magnetic resonance imaging (iMRI) helps to increase the EOR of pituitary adenomas [4,5,6]. Despite the improvement in intraoperative tumor visualization with the endoscopic technique, iMRI has been shown to have a synergistic effect on EOR [2,6,7]. In our study, we evaluated a cohort of patients with recurrent adenomas who received iMRI after adenoma resection and assumed GTR. We analyzed the potential of iMRI to identify resectable tumor remnants and whether iMRI might lead to increased EOR in recurrent adenomas.

## 2. Materials and Methods

### 2.1. Patients and Follow-Up

We performed a retrospective analysis of patients who underwent surgical treatment of recurrent pituitary adenomas at our department between 2013 and 2021. All pituitary surgeries were planned for iMRI-assisted resection at our department. Only in the cases of contraindications for iMRI, surgical emergency, or unavailability of iMRI, a transsphenoidal resection without iMRI was performed. Pituitary adenomas were graded according to the criteria published by Knosp et al. [8]. For a subgroup analysis, less invasive adenomas were defined as Knosp grades 0–2 and invasive adenomas as Knosp grades 3–4. Postoperative assessments of clinical and endocrine status as well as MRI were performed 3 months after surgery; further follow-up was scheduled yearly or every 2 years depending on the histologic adenoma growth patterns and the clinical status. PFS was defined as the time between surgery and the detection of a new suspicious lesion indicative of a recurrent tumor on follow-up MRI, or an increase in postoperative tumor remnant in the case of previous subtotal resection (STR). In the case of hormone-producing tumors, progression was defined as the recurrence of hormone excess.

The endocrinological assessment was performed in cooperation with the local endocrinology department, as described in our previous publications [6,7].

Visual deficits due to the compression of the optic chiasm were evaluated before surgery and during follow-up. Besides neurological examination, perimetric investigation was routinely required before all follow-up visits. Improvement, stable state, and worsening of visual alterations were evaluated.

### 2.2. OR Setup and MRI

An intraoperative 1.5 T MRI scanner (Siemens Espree, Siemens AG, Erlangen, Germany) has been available at our department as a one-room solution since October 2008. The analysis of residual tumor as detected by iMRI was performed on thin slice (2 mm) high-resolution coronal and sagittal T2 and with gadolinium-enhanced T1 images. The use of iMRI led to an increase in the duration of surgery by approximately 45 min. At our department, all eligible cases of pituitary adenomas were scheduled for iMRI-assisted resection. In a minority of cases, iMRI was not performed due to unexpected circumstances such as, for instance, technical issues.

### 2.3. MRI Volumetric Assessment

The volumetric assessment and the evaluation of all MRI images were performed in cooperation with the department of neuroradiology. Coronal T2-weighted turbo spin-echo and coronal and sagittal T1 plain and contrast-enhanced sequences obtained before surgery, intraoperatively, and 3 months after surgery were used for the analysis. In the case of unclear findings, a follow-up scan 1 or 2 years after surgery was used for evaluation. Adenomas were segmented semiautomatically using the software Elements (Brainlab, Munich, Germany). GTR was presumed if no clear adenoma remnant was identified.

### 2.4. Surgical Procedure

A microscopic or an endoscopic transsphenoidal approach was used according to the surgeons’ preference. Four neurosurgeons experienced in pituitary surgery performed all the procedures. The microscopic resection was performed using a direct transnasal paraseptal approach. The endoscopic resection was carried out using a bilateral transnasal transsphenoidal approach with partial resection of the vomer. Rigid 0°, 30°, and 45° Hopkins endoscopes were used during the resection.

The reconstruction of the sella was commonly performed with a fibrin-coated sponge. In the case of cerebrospinal fluid (CSF) leak and large diaphragm opening, an abdominal subcutaneous fat graft was implanted in the defect and covered with a fibrin-coated sponge in a multilayer technique.

### 2.5. Surgical Complications

A detailed assessment of surgical complications was performed. CSF leak was the most common complication and was defined as verified rhinoliquorrhea after surgery, which resulted in surgical revision. Infection was defined as a meningitis with an identified pathogen or a case where antibiotic therapy was started even if no pathogen was found. Hypopituitarism and diabetes insipidus were evaluated separately, as described in earlier publications [6,7]. New neurological deficits and thromboembolic complications during hospital stay were collected and analyzed.

### 2.6. Data Analysis

Statistical analysis was performed using SPSS 26.0 (Lead Technologies, INC, Charlotte, NC, USA). Descriptive statistics, Mann–Whitney U tests, and Fisher exact tests were used for the analysis. Univariate and multivariable regression models evaluating the risk of a residual tumor on the iMRI and further resection were used. The influencing variables were age, adenoma volume, sex, surgical technique, Knosp grade, and adenoma subtype. All variables that achieved a significant difference (*p* < 0.05) in the univariate analysis were included in the multivariable analysis.

The study was conducted according to the international Declaration of Helsinki. The approval of the local ethics committee was obtained.

## 3. Results

### 3.1. General Characteristics

A total number of 59 iMRI-assisted resections of recurrent pituitary adenomas were selected for the analysis. Non-functioning adenomas were the most common adenoma type (N = 42, 71.2%), followed by STH-producing (n = 10, 16.9%) and ACTH-producing adenomas (N = 4, 6.8%). Other adenoma types were found in three patients (5.1%). According to the Knosp grading system, 44.1% of the patients were classified as Knosp grades 0–2 and 55.9% as Knosp grades 3–4 (Table 1). Higher Knosp grades were significantly associated with incomplete resection (*p* = 0.014, n = 23/35, 65.7%). The median patient age was 57 years (range: 31–83 years). Males were significantly more common (*p* < 0.001, Table 1).

### 3.2. Extent of Resection

**Figure 1:**
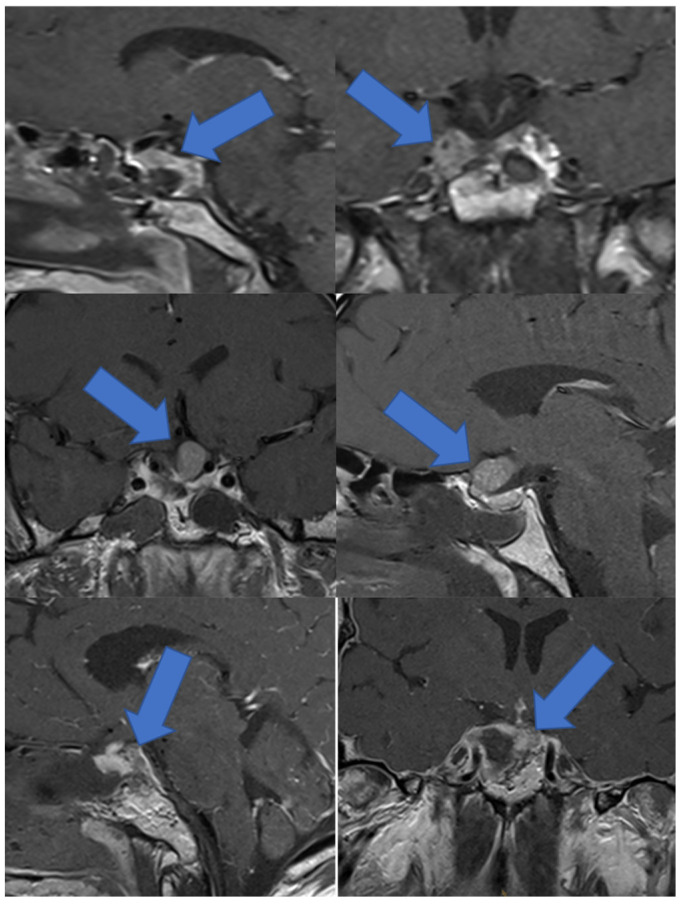
Pictures in the first row depict sagittal and coronal- with gadolinium-enhanced MRI, showing intracavernous tumor remnant. In the second row, tumor remnant perforating the diaphragm is depicted on coronal- and sagittal-enhanced MRI scans, and in the third row, adenoma remnant infiltrating the clivus is shown.

Further resection due to an adenoma remnant found on iMRI was performed in 54.2% of the patient cohort (n = 32). After iMRI, the rate of GTRs increased from 33.9% (n = 20) to 49.2% (n = 29). The median tumor volume on iMRI was 0.4 cm^3^ and the median postoperative tumor volume was 0.02 cm^3^. The microscopic technique was used in 55.9% of cases (n = 33). GTR was achieved with the endoscopic technique in 18 cases (N = 18/26, 69.2%) and was significantly more common (*p* = 0.003) in this instance than with the microscopic technique, where GTR was achieved in 10 cases (N = 10/33, 30.3%). The most common tumor locations amenable to further resection after iMRI were the infiltration of the clivus, the wall of the cavernous sinus, or the perforation of the diaphragm. Examples of tumor remnants found on iMRI are depicted in Figure 1.

According to the Knosp classification, GTR in Knosp grades 0–2 was found in 16 cases (N = 16/24, 66.7%) and in Knosp grades 3–4 in 12 patients (N = 12/35, 34.3%). Interestingly, in the subgroup analysis of adenomas rated Knosp grades 0–2, GTR was achieved in all the endoscopically performed resections. Residual adenoma was found in eight patients in the Knosp grades 0–2 subgroup, all of whom underwent a microscopic adenoma resection. The difference was statistically significant (*p* = 0.014). Furthermore, in Knosp grades 3–4 adenomas, GTR was significantly more common if the endoscopic technique was used (*p* = 0.012, N = 10/18, 55.6% endoscopic technique vs. N = 2/17, 11.8% for the microscopic technique).

In the univariate regression analysis for EOR, a large adenoma volume, the microscopic technique, and a high Knosp grade were significant risk factors for incomplete GTR (Table 2). In the multivariable model, a high Knosp grade and the microscopic technique were statistically significant predictors for incomplete GTR (Table 2). Age and sex showed no significant difference (Table 2).

**Figure 2:**
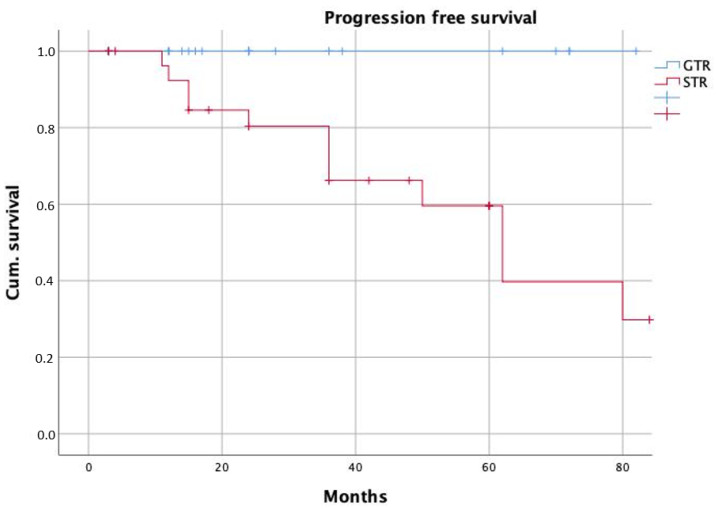
Progression-free survival according to the extent of resection (GTR—gross total resection; STR—subtotal resection).

**Figure 3:**
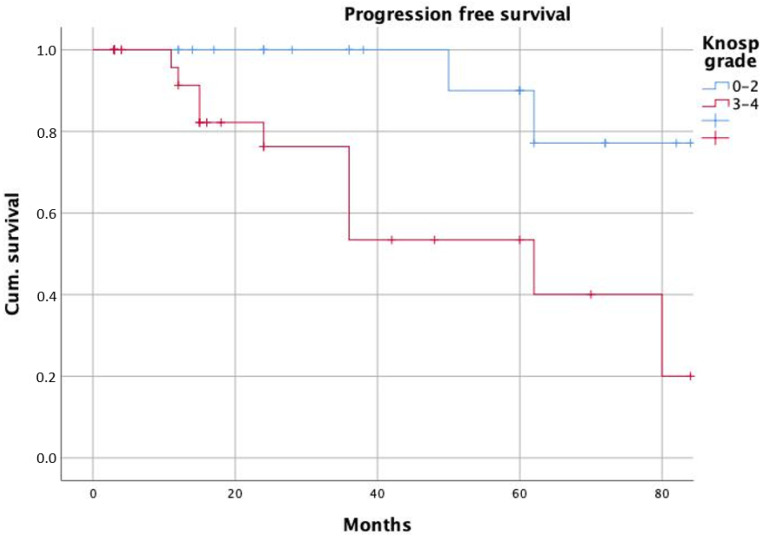
Progression-free survival according to Knosp grades.

The median follow-up time was 24 months. After GTR, patients showed a significantly longer progression-free survival if compared to STR (*p* = 0.005 with long-rank test, Figure 2). Similarly, we found significantly longer PFS in patients with adenoma Knosp grades 0–2 (*p* = 0.002, Figure 3).

### 3.3. Additional Resection of Tumor Remnant Identified on iMRI

A large tumor volume and the microscopic technique were significantly associated with further resection after iMRI in the univariate analysis (Table 3). Patient age, sex, and Knosp grade did not achieve a statistically significant difference (Table 3). Only increasing tumor volume was found to be an independent predictor (Table 3) for further resection. There was a trend towards significance for the microscopic technique (Table 3).

### 3.4. Endocrine Outcome and Visual Improvement

A new hypopituitarism for one or more pituitary axes was found in 12.7% of the patients (N = 7/55), five (15.1%) of whom were treated microscopically vs. two (7.7%) with endoscopic surgeries (*p* = 0.439). Visual disturbances before surgery were identified in 32% of the patients (N = 19). New diabetes insipidus was found in six patients (10.2%) and was always associated with the microscopic technique (*p* = 0.01). Five of these cases (83.3%) were classified as Knosp grade 4; one case was classified as Knosp grade 2 (16.7%). Visual improvement was found in 68.4% of patients (N = 13) after surgery.

Biochemical remission was achieved in eight of the patients with functional adenomas (N = 8/14, 57.1%). Out of the six patients who did not achieve biochemical remission, three had invasive adenoma Knosp grade 4 and three had no visible tumor remnant on MRI.

### 3.5. Complications

Meningitis was diagnosed in one patient (1.7%). Surgical revision was performed in two cases (3.4%) due to CSF leak. In both cases, the procedure was performed with the microscopic technique. In two cases (3.4%), oculomotor nerve palsy was found after surgery but resolved completely 3 months after the operation. One patient suffered from symptomatic pulmonary embolism during their hospital stay and needed anticoagulation therapy, but recovered completely without further complications.

### 3.6. Illustrative Case

**Figure 4:**
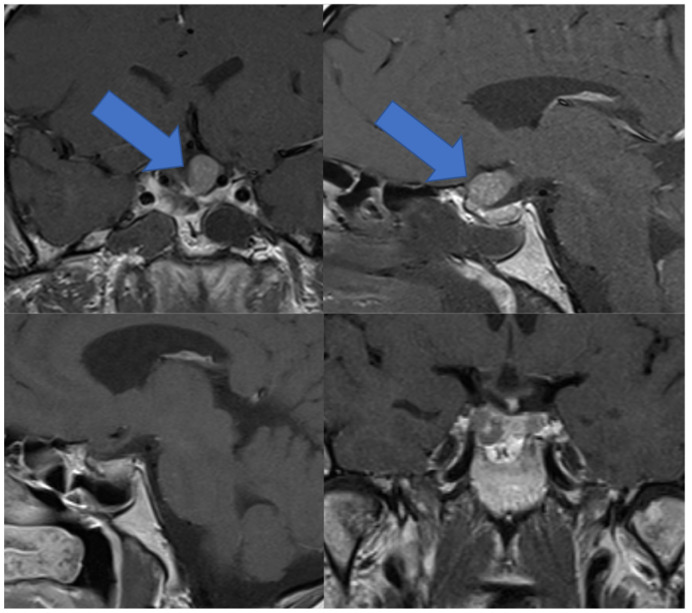
Intraoperative MRI depicting adenoma remnant and follow-up MRI 3 months after surgery showing decompressed chiasm and fat graft used for reconstruction of the sella.

A 38-year-old male showed progress of recurrent adenoma and optic atrophy of the left optic nerve. An endoscopic iMRI-assisted transsphenoidal resection was performed. After, an adenoma resection exploration of the diaphragm folds was performed and no clear penetration was found. iMRI was performed to confirm GTR. iMRI and postoperative MRI are depicted in Figure 4. An intradural tumor remnant was identified; consequently, an intentional incision of the diaphragm was performed, and the recurrent adenoma was separated from the optic nerve and resected. An abdominal subcutaneous fat graft was used for the reconstruction of the sella. The MRI images after surgery are depicted in Figure 4.

## 4. Discussion

Limited evidence is available regarding the optimal choice for the treatment of recurrent pituitary adenomas [9]. Repeated resection, stereotactic radiosurgery, fractionated radiation therapy, fractionated stereotactic radiotherapy and intensity-modulated radiotherapy are possible treatment modalities. For non-functioning adenomas, there is a level II recommendation for radiosurgery and radiation therapy for the treatment of residual or recurrent tumors [10]. Furthermore, level III evidence exists for the repeated resection of symptomatic recurrent non-functioning adenomas. Compared to radiosurgery or radiotherapy, recurrent surgery seems to have a favorable complication profile [9,11,12]. The extent of resection can be limited due to the invasiveness of the tumor and the distortion of anatomic landmarks as a consequence of previous surgery. Chang et al. noted a substantial discordance between the intraoperative impression of the surgeon and the postoperative imaging regarding the extent of resection in 39% of cases [11]. Some of these difficulties could be overcome with an objective intraoperative assessment of the actual extent of resection.

Intraoperative visualization modalities, such as iMRI, have been shown to have a positive impact on the EOR in various intracranial pathologies [13,14,15,16]. As reported by Coburger et al. in our earlier analysis, iMRI significantly increased the number of GTRs and decreased the residual tumor volumes of adenomas after microscopic resection when compared to a historical cohort treated without iMRI [17]. Here, we evaluated the possible benefit of iMRI on transsphenoidal resections of recurrent pituitary adenomas. The use of iMRI significantly increased the EOR and the number of GTRs. Furthermore, the complication rate was low with a high number of patients with an improvement of visual disturbances and a very low number of patients who needed revision surgery. To our knowledge, no published work so far has analyzed the impact of iMRI on surgery for recurrent pituitary adenomas. Despite the significant increase in the EOR, our results are clearly not superior compared to recent publications on patients treated without iMRI [12,18]. This may be explained by the relatively high proportion of Knosp grade 3 and 4 tumors in our cohort. In the endoscopically treated tumors with Knosp grades 0–2, the GTR rate was 100% in our analysis and 68.4% in the analysis of Do et al. [12]. In the invasive tumors of Knosp grades 3–4, the GTR rate in our study was 55.6% vs. 21.7% in the study of Do et al. The multivariable regression model showed that the increasing tumor volume was significantly associated with tumor remnants on the iMRI which were amenable to further resection. This was not the case in the multivariable model for GTR. A potential explanation is the possibility to decrease the impact of pure tumor volume by iMRI visualization, so that the treatment of especially large tumors may benefit from iMRI. Davies et al. have analyzed the quantitative assessment of tumor volume and compared it with qualitative spread beyond the anatomical borders as defined by Knosp et al.: they suggested that both methods provide complementary information which may be helpful for further treatment strategy. Even if the invasion of the cavernous sinus is extremely relevant to the resection, the tumor volume is still an important parameter even though it was not confirmed as an independent predictor in our multivariable analysis. The iMRI technique might help for reaching adenoma remnants hidden in the diaphragm folds, perforating the diaphragm or invading the cavernous sinus [6,19].

Our findings also point out the superiority of the endoscopic approach with regard to GTR, as was the case in the meta-analysis of Equenazi et al., who compared 292 patients treated endoscopically with 648 patients treated microscopically. They found a small difference in the overall GTR of 53.5% in the endoscopic treatment group versus 46.6% in the microscopic treatment group. However, an inhomogeneity between these groups was found with significantly more non-functioning macroadenomas and a significantly higher proportion of cavernous sinus invasions in the endoscopic treatment cohort.

GTR has been shown to have a positive impact on PFS in pituitary adenoma surgery patients [2]. Our analysis underlines this aspect also for recurrent adenomas. Similarly, patients with less aggressive invasion of the cavernous sinus showed better PFS.

The rate of new pituitary insufficiency after surgery was comparable with other studies, especially if we take into consideration that our cohort included only recurrent adenomas. The endoscopic technique seems to be beneficial with regard to diabetes insipidus [20]. Furthermore, the number of iatrogenic pituitary insufficiency cases was higher with the microscopic technique even though the difference was not statistically significant. This result is congruent with recently published studies [6,20]. The endoscopic technique potentially leads to a better and earlier visualization and identification of the pituitary gland, which results in a more precise dissection and the preservation of function [21,22].

Gamma knife radiosurgery has been routinely established in the treatment of recurrent adenomas. According to a meta-analysis published by Albano et al., tumor control is up to 93% and the 5-year PFS, 95% [23]. However, suprasellar extension and a large tumor volume were suggested as negative predictors for PFS after radiation therapy [24]. Furthermore, the rates of new pituitary insufficiencies after radiation therapy are still high. Therefore, surgical resection enhanced by improved visualization using the endoscopic technique and reevaluation by iMRI may relevantly reduce tumor volume and create a buffer zone between the pituitary gland, the optic chiasm, and tumor remnants. This could dramatically reduce the risk of complications of radiosurgery [23]. This effect seems to be even more relevant for functional adenomas with defined morphological correlates in MRI. Compared to the radiation therapy of non-functional adenomas, these tumors usually require higher doses in the gamma knife treatment in order to achieve biochemical remission, especially in the areas of the tumor margins [23].

### Limitations

The retrospective design, the relatively small number of cases, and the lack of a control group treated without iMRI are the main limitations of our study. Due to the improvements of the EOR during the primary surgery for pituitary adenomas and the slow growth of the tumors, the number of operations for recurrent adenomas is generally low, which makes it difficult to create a prospective study design. iMRI was performed after the surgeon presumed complete or maximal safe resection. Surely, this may lead to the bias that the EOR was influenced by the possibility of an additional resection of a tumor remnant visible on iMRI. It is very difficult to define a standardized and objective trigger for iMRI during tumor resection. Based on our experience, a reliable though subjective trigger in the hands of an experienced surgeon is the point when further dissection would appear to substantially increase the risk of surgery. iMRI is an expensive tool for intraoperative visualization with limited availability and it is difficult to calculate the cost effectiveness from our data. Further studies are needed to resolve this question.

## 5. Conclusions

According to our data, patients with a large recurrent adenoma benefit most from iMRI-assisted transsphenoidal resection. The positive influence of iMRI was more pronounced with the microscopic technique. The employment of the endoscopic technique resulted in higher resection rates and lower rates of new postoperative pituitary insufficiencies. Furthermore, occult invasions of the cavernous sinus and the clivus were the most common findings leading to the further resection of tumor remnants after iMRI.

## Figures and Tables

**Table 1:** Patients’ and tumor characteristics.

Patient and Tumor Characteristics	Total
n	59
Age (Median)	57
Male Ratio	71.2% (42)
Median Tumor Volume (cm^3^)	3.3
Gross Total Resection	49.2% (29)
Non-Functioning Adenoma	71.2% (42)
Knosp Grades 3–4	55.9% (33)
Endoscopic Technique	44.1% (26)
Microscopic Technique	55.9% (33)
Adenoma Remnant Resection after iMRI	54.2% (32)

**Table 2:** Univariate and multivariable analyses of factors with potential impact on gross total resection.

Variables	Univariate Analysis			Multivariable Analysis		
	HR	95% CI	*p*	HR	95% CI	*p*
Age	1.0	0.9–1.1	0.067			
Sex	1.4	0.4–4.2	0.592			
Knosp grade	3.8	1.3–11.5	0.017	6.6	1.2–36.1	0.031
Surgical technique	5.2	1.7–15.8	0.004	6.2	1.1–34.9	0.038
Tumor volume	1.5	1.1–1.9	0.005	1.2	1.0–1.3	0.063

**Table 3:** Univariate and multivariable analyses of factors which resulted in incomplete resection and tumor remnants in intraoperative MRI.

Variables	Univariate Analysis			Multivariable Analysis		
	HR	95% CI	*p*	HR	95% CI	*p*
Age	1.0	0.98–1.1	0.255			
Sex	0.7	0.2–2.5	0.660			
Knosp grade	2.4	0.8–7.0	0.119			
Surgical technique	4.4	1.4–13.7	0.009	4.3	0.97–19.4	0.054
Tumor volume	1.6	1.2–2.1	0.004	1.5	1.1–2.1	0.007

## Data Availability

The data presented in this study are available on request from the corresponding author.
